# The relationship between distress tolerance and behavioral activation on anxiety and depression symptomatology in autistic youth: Leveraging self and caregiver perspectives

**DOI:** 10.1002/aur.3208

**Published:** 2024-08-06

**Authors:** Jessica M. Schwartzman, Ligia Antezana, Caitlin M. Conner

**Affiliations:** 1 Department of Psychiatry and Behavioral Sciences, Vanderbilt University Medical Center, Nashville, Tennessee, USA; 2 Department of Pediatrics, University of Southern California, Los Angeles, California, USA; 3 Department of Psychiatry, University of Pittsburgh School of Medicine, Pittsburgh, Pennsylvania, USA

**Keywords:** adolescent, anxiety, autism, behavioral activation, depression, distress tolerance

## Abstract

Anxiety and depression are prevalent among autistic adolescents and may be difficult to accurately diagnose and treat given various factors (e.g., diagnostic overshadowing, heterogeneity). Therefore, efforts to examine transdiagnostic factors (i.e., distress tolerance, behavioral activation) may afford more parsimonious means for assessment and treatment. To our knowledge, there has been little research on distress tolerance, behavioral activation, and depressive and anxiety symptoms in autistic adolescents to guide diagnostic practices and treatment planning. In the current study, we examined the interrelationships between these transdiagnostic factors and depressive and anxiety symptoms using ratings from 100 verbally fluent autistic adolescents without intellectual disability (*M*_age_ = 13.70, SD_age_ = 2.23, Range: 11:00–17:11 years) and 100 of their caregivers. Many adolescents reported male sex assigned at birth (61%), cisgender (87%), not Hispanic/Latinx (90%), and White (80%) identities. A series of correlational analyses were employed to examine associations between these constructs from youth and caregiver perspectives, and multiple linear regression analyses were conducted to explore the mediating roles of distress tolerance and behavioral activation. Preliminary results show that low distress tolerance and behavioral activation were associated with more severe internalizing symptoms per self- and caregiver-report. Some differences by rater emerged, which highlight the importance of multi-informant ratings in autism. Results from mediation analyses may show that behavioral activation may be more salient to assessments and treatment planning for depression than distress tolerance, while distress tolerance may be important for both anxiety and depression; however, findings are preliminary given the cross-sectional nature of the data. Findings suggest that these transdiagnostic concepts may be important to individualizing treatment approaches, including the timing of certain approaches, for anxiety and/or depression in autistic adolescents.

## Lay Summary

Anxiety and depression are common among autistic adolescents and may be difficult to accurately diagnose and treat. Therefore, efforts to examine transdiagnostic factors (i.e., shared traits across mental health conditions), such as distress tolerance and behavioral activation, may afford more parsimonious means for assessment and treatment. In the current study, we examined the interrelationships between distress tolerance, behavioral activation, and depressive and anxiety symptoms using ratings from 100 autistic adolescents (11:00–17:11 years) and 100 of their caregivers. Results show that low distress tolerance and behavioral activation were associated with more severe anxiety and depressive symptoms per self- and caregiver-report. Findings also show that behavioral activation may be more relevant to treatment planning for depression than distress tolerance, while distress tolerance may be important for both anxiety and depression.

## INTRODUCTION

Mental health distress is highly prevalent in autistic adolescents, with up to 70% of autistic adolescents in a community-sourced sample meeting criteria for any DSM-5 disorder (Hollocks et al., 2022) and 41% of youth meeting criteria for more than one psychiatric condition (Simonoff et al., 2008). Given this high prevalence and the reported difficulties in diagnosing psychiatric conditions in autistic people (e.g., diagnostic overshadowing; Lai et al., 2019), examining *transdiagnostic factors* (i.e., distress tolerance, behavioral activation) may afford more parsimonious means for assessment and treatment. One of the most researched transdiagnostic concepts in autism is emotion regulation (ER; i.e., one’s ability to modulate the cognitive, behavioral, and physiological aspects of affect; Bargh & Williams, 2007; Rottenberg & Gross, 2007). Difficulties with ER are up to four times more likely in autistic adolescents than non-autistic adolescents (Conner et al., 2021), and poor ER is associated with psychiatric conditions in autistic people (Cai et al., 2018; Charlton et al., 2020; Conner et al., 2023; Mazefsky, 2015).

Two transdiagnostic factors which are related, but distinct constructs from ER are distress tolerance (DT) and behavioral activation (BA; Aldao et al., 2012; Mennin et al., 2005). DT is defined as one’s ability to endure negative emotional or aversive states (Zvolensky et al., 2010), and is frequently targeted in interventions such as Dialectical Behavior Therapy (DBT; Linehan, 1987) or other ER-focused treatments for autistic people (Conner et al., 2019; Scarpa et al., 2012; Shaffer et al., 2023). Distress tolerance broadly includes constructs of tolerance of uncertainty, ambiguity, frustration, negative emotion, and physical discomfort (Zvolensky et al., 2010). Distress tolerance has also been conceptualized as a type of emotion regulation strategy, and has been most commonly studied in the context of high-risk behaviors such as self-injury, suicidality, and substance abuse, although it has also been found to be associated with depression and anxiety (Conway et al., 2021). Often, measures assessing tolerance of specific cognitive constructs (e.g., uncertainty, ambiguity) are conceptualized under the umbrella of DT (Leyro et al., 2010). Intolerance of uncertainty (IU) is a transdiagnostic construct defined as difficulty or inability to tolerate the effects caused by a lack of information in a given situation, whether a fear of future events or present-based fears of uncertainty (Carleton, 2016). Intolerance of uncertainty has been conceptualized as a component of DT, although IU has been primarily previously studied in reference to anxiety disorders, unlike DT. Further, DT includes other components, such as tolerance of physical and emotional discomfort (Zvolensky et al., 2010). Studies have shown that high levels of IU are more prevalent in autistic people than non-autistic people (Boulter et al., 2014; Vasa et al., 2018), and IU is similarly correlated with anxiety and depression in autistic people (Conner et al., 2022; Keefer et al., 2018). Therefore, an understanding of DT in the pathway to internalizing symptoms in autistic adolescents is a critical extension of this work as internalizing disorders are common in autistic adolescents, DT is a risk factor for internalizing disorders, and research on DT in autistic adolescents is limited.

BA is prioritizing and engaging in meaningful activities to decrease avoidant behaviors and improve mood (Kanter et al., 2010), and has primarily been studied in the context of intervention for depression, although it is linked to internalizing disorders more broadly (Kanter et al., 2010; Stein et al., 2021). Emerging research in autistic adolescents and adults shows that low BA is associated with more severe internalizing symptoms and may be an important intervention target (Bal et al., 2023; Schwartzman & Bonner, 2023), with initial efficacy in treating depressive symptoms in youth in a pilot nonrandomized trial (Menezes et al., 2024). Despite this emerging research, the evidence base on BA in autistic people is limited. While transdiagnostic constructs strongly overlap with internalizing disorders such as depression and anxiety, they do not explain all of the variance in either diagnosis (Conway et al., 2021). Neither DT nor BA have been widely studied in autistic samples. Given the broad nature of ER and these related transdiagnostic constructs, it warrants continued investigation to understand whether interrelated concepts and specific facets function similarly in autistic people in association with mental health distress.

Previous work in non-autistic samples examining both DT and ER revealed a complex relationship with potentially tighter interrelationships between these constructs in individuals with greater emotional and behavioral difficulties (Conway et al., 2021; Van Eck et al., 2017). For example, lower DT and ER abilities both contribute to greater avoidance-based coping (McHugh et al., 2013). Moreover, in adults with poorer attentional control, there was a relationship between difficulties in accessing effective ER strategies and DT (Bardeen et al., 2015). Further, individuals with low DT may demonstrate more “maladaptive” ER strategies (e.g., rumination, avoidance, emotion suppression) than individuals with high DT (Jeffries et al., 2016; Naragon-Gainey et al., 2017). Although this literature base does not specifically examine BA, patterns with avoidance, rumination, and less access to effective ER may imply important interrelationships between DT and BA. As noted, low DT may lead to coping behaviors that reduce distress quickly, but ultimately prove to be problematic coping behaviors (e.g., avoidance, rumination); this may explain how one’s ability to tolerate distress may contribute to and maintain internalizing symptoms (Lass et al., 2020; Lass & Winer, 2020). As DT, BA, and internalizing symptoms are highly connected in the non-autistic population, it is important to consider these interrelationships in autistic adolescents, as targeting these transdiagnostic factors could also improve treatment effectiveness and precision treatment approaches for autistic people.

In autistic populations, sex and gender differences in ER, intolerance of uncertainty, anxiety, and depression and their relationships have been documented (Jenkinson et al., 2020; Oswald et al., 2016; Wieckowski et al., 2020). Despite an increasing focus on ER in autistic individuals (Cai et al., 2018), few studies have specifically examined the broad construct of DT or BA in autism (Eldeeb et al., 2021; Schwartzman et al., 2023) and recent work has highlighted this need (Bal et al., 2023; Rette et al., 2021). Though autistic people assigned female at birth may be more likely to experience internalizing disorders than people assigned male at birth (Cai et al., 2017; Jenkinson et al., 2020; Oswald et al., 2016; Wieckowski et al., 2020), sex and gender differences in transdiagnostic factors (e.g., distress tolerance, behavioral activation) remain untested in this population and may be critical to guiding the development of future studies and potential interventions.

The present study investigated relationships between DT, BA, and internalizing symptom severity in a clinical sample of verbally fluent autistic adolescents without intellectual disability from both adolescent and caregiver perspectives at one timepoint. Given the cross-sectional nature of the study, data analyses and interpretation are considered preliminary and should be interpreted with caution. It was expected that lower BA and DT would be associated with more severe internalizing symptoms across raters. We anticipated sex-based differences such that autistic adolescents assigned female at birth would endorse lower DT and BA, and more severe internalizing symptoms, than their peers assigned male at birth. Additionally, we explored the potential mediating effects of DT and BA on associations with internalizing symptoms using separate models for adolescent and caregiver ratings.

## METHODS

### Study design

The present study was a retrospective analysis of de-identified data collected at the same timepoint from a clinical sample of autistic adolescents and their caregivers. As noted, given the cross-sectional nature of the data collected and analyzed in this study, findings should be interpreted as preliminary and with caution.

### Participants

Autistic adolescents between 11:00 and 17:11 years old and one of their caregivers participated in an intake appointment for psychological services (e.g., diagnostic evaluation, psychotherapy) at Vanderbilt University Medical Center. Families sought various psychological services including first-time autism diagnostic evaluations, psychological evaluations for diagnostic clarity and treatment planning, and psychotherapy for anxiety, depression, or other internalizing conditions. All adolescents were diagnosed with autism previously by a qualified mental health professional or by the clinicians. Existing autism diagnoses were validated by review of previous testing reports and medical records to confirm autism diagnostic status and methods (e.g., administration of the Autism Diagnostic Observation Schedule, Second Edition, ADOS-2 in the testing battery, regardless of outcome as scores are not always available in reports; Lord et al., 2012). Further, estimates of an adolescent’s intellectual abilities (e.g., Full Scale IQ at/above 70 on the Wechsler Abbreviated Scale of Intelligence, Fifth Edition) were collected from record review. New autism diagnoses were established using clinical interviews, clinical judgment, and a specialized assessment battery that included the ADOS-2 (Lord et al., 2012), with intellectual abilities estimated from standardized cognitive assessments (e.g., Full Scale IQ at/above 70 on the WISC-V).

Although the clinic serves a variety of neurodivergent adolescents and adults with different communication, adaptive, and cognitive abilities, several inclusion/exclusion criteria were applied in the present study. Specifically, adolescents met the following inclusion criteria: (1) 11:00–17:11 years old, (2) with Autism Spectrum Disorder, (3) without intellectual disability, per medical record review, to complete the self-report measures, (4) comfortable reading and writing in English to complete self-report questionnaires, and (5) a caregiver available to complete questionnaires about adolescent mental health.

### Procedure

As part of standard clinical services, families provided verbal and written consent/assent for these services, which included the use of adolescent self- and caregiver-report questionnaires. All families presenting to the clinic for services provided clinical data that could be de-identified and used for research purposes with approval from the Institutional Review Board; however, in the present study as described, de-identified clinical data were extracted only from youth who met the inclusion/exclusion criteria for the present study. De-identified data from questionnaires administered during intake appointments between January 2021 and December 2022 were analyzed in the present study in accordance with approval from the Institutional Review Board at Vanderbilt University (Protocol #211870). Research procedures to extract de-identified data from the clinical intake battery for research purposes were approved by the IRB and thus, a waiver of consent was approved.

### Measures

#### Demographic information

Adolescents and caregivers each completed demographic forms (see Table 1). Information about adolescent psychiatric services was collected including medication status, previous/current counseling, previous psychiatric hospitalization, and aggression to self and/or others in the past 6 months. Caregivers reported their highest level of education, current employment status, and annual household income. Families had the opportunity to select the option, “Prefer not to say,” for all demographic questions. Parents completed the Social Responsiveness Scale, Second Edition (SRS-2; Constantino & Gruber, 2012) to assess autistic traits in the past 6 months.

#### Revised Children’s Anxiety and Depression Scale

The Revised Children’s Anxiety and Depression Scale, Parent and Child versions (RCADS-P and RCADS-C, respectively; Chorpita et al., 2005) is a caregiver- and self-report questionnaire that assesses the severity of adolescent internalizing symptoms in the past 2 weeks. The RCADS-P/C has been validated for use with autistic adolescents (Kaat & Lecavalier, 2015; Sterling et al., 2015). In the present study, T-scores from the depression and total anxiety subscales of the RCADS-P and RCADS-C were included in analyses and demonstrated acceptable and good internal consistency, respectively (Depression: caregiver *α* = 0.78, adolescent *α* = 0.82; Anxiety: caregiver *α* = 0.92, adolescent *α* = 0.96).

#### Behavioral Activation for Depression Scale-short form

The Behavioral Activation for Depression Scale-Short Form (BADS-SF; Manos et al., 2011) is a 9-item questionnaire that assesses the level of behavioral activation and avoidance in the previous week, with particular focus on meaningful activities. Items are summed to generate a total score with higher scores suggesting a higher level of behavioral activation, and vice versa. The total scores from adolescent and caregiver ratings on the BADS-SF were used in the analyses of the present study and demonstrated good internal consistency (caregiver *α* = 0.80; adolescent *α* = 0.82).

#### Distress Intolerance Index

The Distress Intolerance Index (DII; McHugh & Otto, 2012) is a 10-item questionnaire that assesses one’s perceived distress intolerance (i.e., inability to tolerate negative somatic and emotional states; see review by Zvolensky et al., 2010). Items are summed for a total score of DT, with higher scores indicating higher DT and lower scores indicating lower DT. To understand caregiver perspectives, a caregiver-report version of the DII was created and administered to caregivers. The total scores from adolescent and caregiver ratings on the DII were used in the analyses of the present study and demonstrated excellent internal consistency (caregiver *α* = 0.92; adolescent *α* = 0.94).

### Statistical analysis

Data analyses were performed in SPSS Version 28. Descriptive statistics were calculated for sample characterization and to identify any outliers (i.e., any participant with scores >3 *SD* from the sample mean), and no cases were removed. To understand adolescent and caregiver perspectives on study variables (i.e., DT, BA, internalizing symptom severities), a series of paired samples *t*-tests were employed as adolescents and caregivers completed the same questionnaires. A series of correlational analyses were employed to examine associations between adolescent DT, BA, and internalizing symptom severity from adolescent and caregiver perspectives. Not all continuous variables were normally distributed, as assessed by Kolmogorov–Smirnov statistic tests (*p* < 0.05). Therefore, Spearman’s rho correlations were employed. Correlation coefficients were interpreted following guidelines from Dancey and Reidy (2011): weak (0.1–0.3), moderate (0.4–0.6), strong (0.7–0.9), and perfect (1.0; Dancey & Reidy, 2011). Sex differences on study variables were tested using Mann–Whitney *U* tests or *t*-tests for independent samples, depending on whether assumptions for parametric tests were met. Separate analyses were conducted for adolescent self- and caregiver-reported data given rater differences (see Supplemental Tables 1 and 2). If adolescent or caregiver demographic variables were associated with study variables, these were accounted for in the subsequent mediation analyses.

To explore the mediating role of DT in the relationship between BA and depressive and anxiety symptom severities, separate linear regression analyses were conducted in the PROCESS macro for SPSS (Hayes, 2018). Covariates were added into the models, as appropriate. Standardized effect sizes were reported as these may have less bias than proportion- and ratio-based methods (Miočević et al., 2018). Mediation analyses were conducted for adolescent and caregiver ratings for both depressive and anxiety symptom severities. A similar mediation model was conducted, as described above, with BA as a potential mediator of the association between DT and depressive and anxiety symptom severities.

## RESULTS

### Sample characterization

Table 1 presents demographic information for the 100 autistic adolescents and 100 caregivers. Adolescents were between 11:00 and 17:11 years old (*M* = 13.70, SD = 2.23), verbally fluent, and without intellectual disability. The majority of adolescents reported male sex assigned at birth (61%), cisgender boy/girl (87%), not Hispanic/Latinx (90%), and White (80%) identities. Most adolescents were prescribed psychiatric medications (76%) and received psychotherapy (78%). A proportion of adolescents (30%) were previously hospitalized for psychiatric reasons. Some adolescents exhibited aggression to self (35%) and others (28%) at the time of intake. On average, adolescent Total *T*-scores on the SRS-2 were in the Moderate range (*M* = 73.61, SD = 11.68, range: 60–90). The study sample, and clients served by the Vanderbilt clinic, are not reflective of many autistic adolescents with diverse identities; these are areas for improvement in our research and clinical practices.

Caregivers were between 32 and 78 years old (*M* = 45.27, SD = 7.19), with the majority reporting female sex (90%), cisgender identity (89%), not Hispanic/Latinx (94%), and White (85%) identities. Most caregivers had college (45%) or graduate degrees (28%) and were employed (66%). The majority of families (54%) had an annual household income at or above $75,000 USD. Families possessed health insurance plans that covered behavioral health services, which does not reflect the experiences of all autistic adolescents and their families.

### Associations between demographic and study variables by rater

Adolescent age was not associated with ratings of DT, BA, nor internalizing symptom severities (see Table 2). Sex differences emerged in adolescent ratings of DT (*U* = 1034.50, *z* = 2.74, *p* = 0.006) and anxiety symptom severity (*U* = 1128.00, *z* = 2.44, *p* = 0.015), as well as caregiver ratings of adolescent anxiety symptoms (*U* = 1207.50, *z* = 2.90, *p* = 0.004). Results from pairwise comparisons revealed higher ratings of anxiety symptom severity and DT among autistic adolescents assigned female at birth. Adolescent and caregiver ratings of BA and depressive symptom severity did not differ between the sexes.

Caregiver age, sex, education level, and annual household income level were not associated with ratings of adolescent DT, BA, nor internalizing symptom severities. As differences in DT and anxiety symptom severity were observed between the sexes, sex was included as a covariate in these subsequent mediation analyses.

### Associations between DT, BA, and internalizing symptom severities

Results from Spearman’s rho correlations are presented in Table 2. Similar patterns emerged across adolescent and caregiver ratings. Distress tolerance was negatively associated with depressive and anxiety symptom severities such that adolescents with lower DT exhibited more severe internalizing symptoms, and vice versa. Additionally, DT was positively associated with BA such that adolescents with lower DT endorsed lower BA, and vice versa. Behavioral activation was negatively associated with depressive and anxiety symptom severities such that adolescents with lower BA reported more severe internalizing symptoms, and vice versa, across both raters.

### Mediation analyses: Depressive symptom severity

#### Caregiver-report

**FIGURE 1:**
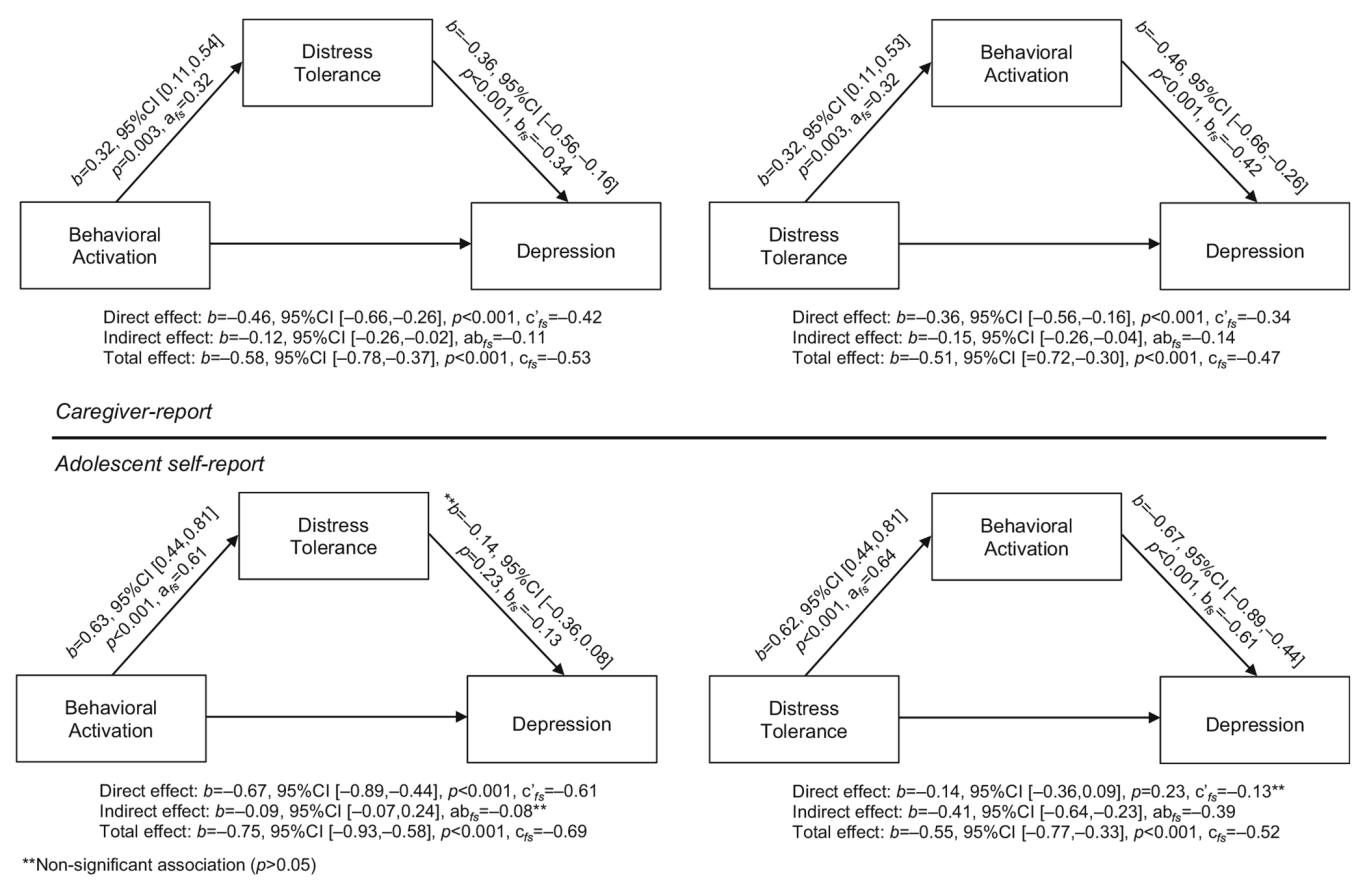
Mediation models of behavioral activation (BA) and distress tolerance (DT) on depressive symptom severity, per caregiver- and adolescent self-report.

Results from mediation analyses are presented in Figure 1; as noted, given the cross-sectional nature of the data collected and analyzed, results should be interpreted as preliminary and with caution. The first analysis examined whether DT mediated the relationship between BA and depressive symptom severity, per caregiver-report, while including sex as a covariate. Significant associations emerged between BA and DT (*b* = 0.32, 95%CI (0.11,0.54), *a_fs_* = 0.32, *p* = 0.003) and between DT and depressive symptom severity (*b* = −0.36, 95%CI (−0.56,−0.16), *b_fs_* = −0.34, *p* < 0.001). The total effect of BA on depressive symptom severity was significant (*b* = −0.58, 95%CI (−0.78, −0.37), *c_fs_* = −0.53, *p* < 0.001), as well as the direct effect (*b* = −0.46, 95%CI (−0.66, −0.26), *c’_fs_* = −0.42, *p* < 0.001). The indirect effect of DT on the relationship between BA and Depressive symptom severity was also significant (*b* = −0.12, 95%CI (−0.26,−0.02), *ab_fs_* = −0.11), with 20.69% of the total proportion mediated.

The second analysis examined whether BA mediated the relationship between DT and depressive symptom severity, per caregiver-report. Significant associations emerged between DT and BA (*b* = 0.32, 95%CI (0.11,0.53), *a_fs_* = 0.32, *p* = 0.003) and between BA and depressive symptom severity (*b* = −0.46, 95%CI (−0.66,−0.26), b_*fs*_ = −0.42, *p* < 0.001). The total effect of DT on depressive symptom severity was significant (*b* = −0.51, 95%CI (−0.72,−0.30), *c_fs_* = −0.47, *p* < 0.001), as well as the direct effect (*b* = −0.36, 95%CI (−0.56,−0.16), *c’_fs_* = −0.34, *p* < 0.001). The indirect effect of BA on the relationship between DT and depressive symptom severity was significant (*b* = −0.15, 95%CI (−0.26,−0.04), *ab_fs_* = −0.14), with 27.45% of the total proportion mediated.

#### Adolescent self-report

The third analysis examined whether DT mediated the relationship between BA and depressive symptom severity, per adolescent self-report. Significant associations emerged between BA and DT (*b* = 0.63, 95%CI (0.44,0.81), *a_fs_* = 0.61, *p* < 0.001), but not between DT and Depressive symptom severity (*b* = −0.14, 95%CI (−0.36,0.08), b_*fs*_ = −0.13, *p* = 0.23). The total effect of BA on Depressive symptom severity was significant (*b* = −0.75, 95%CI (−0.93,−0.58), *c_fs_* = −0.69, *p* < 0.001), as well as the direct effect (*b* = −0.67, 95%CI (−0.89,−0.44), *c’_fs_* = −0.61, *p* < 0.001). The indirect effect of DT on the relationship between BA and Depressive symptom severity was not significant (*b* = −0.09, 95%CI (−0.07,0.24), *ab_fs_* = −0.08).

The fourth analysis examined whether BA mediated the relationship between DT and depressive symptom severity, per adolescent self-report. Significant associations emerged between DT and BA (*b* = 0.62, 95%CI (0.44,0.81), *a_fs_* = 0.64, *p* < 0.001) and between BA and depressive symptom severity (*b* = −0.67, 95%CI (−0.89,−0.44), *b_fs_* = −0.61, *p* < 0.001). The total effect of DT on depressive symptom severity was significant (*b* = −0.55, 95%CI (−0.77,−0.33), *c_fs_* = −0.52, *p* < 0.001); however, the direct effect was not significant (*b* = −0.14, 95%CI (−0.36,0.09), *c’_fs_* = −0.13, *p* = 0.23). The indirect effect of BA on the relationship between DT and depressive symptom severity was significant (*b* = −0.41, 95%CI (−0.64,−0.23), *ab_fs_* = −0.39), with 70.90% of the total proportion mediated.

### Mediation analyses: Anxiety symptom severity

#### Caregiver-report

**FIGURE 2:**
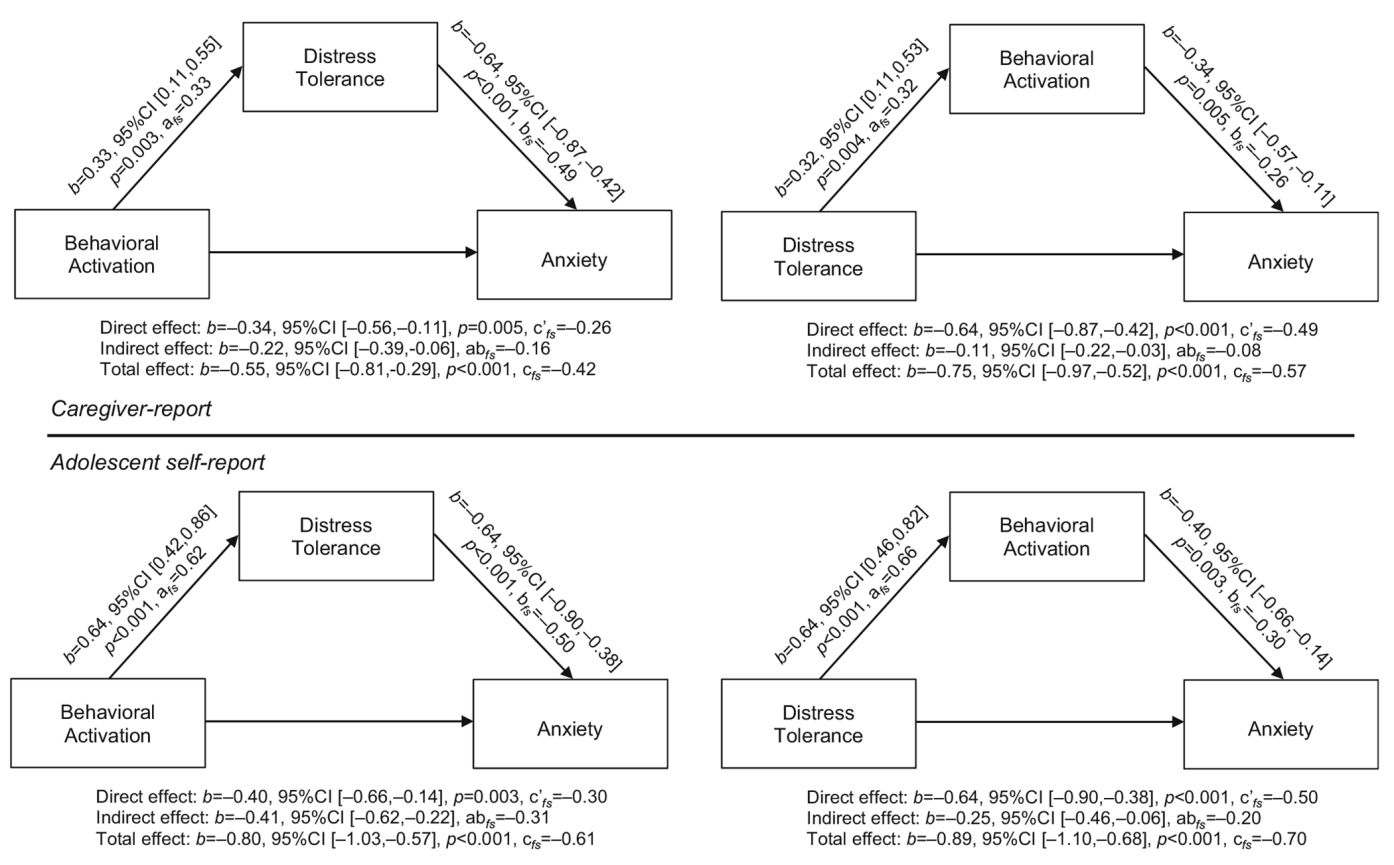
Mediation models of behavioral activation (BA) and distress tolerance (DT) on anxiety symptom severity, per caregiver- and adolescent self-report.

Results from mediation analyses are presented in Figure 2. The fifth analysis examined whether DT mediated the relationship between BA and anxiety symptom severity, per caregiver-report, while including sex as a covariates. Significant associations emerged between BA and DT (*b* = 0.33, 95%CI (0.11,0.55), *a_fs_* = 0.33, *p* = 0.003) and between DT and anxiety symptom severity (*b* = −0.64, 95%CI (−0.87,−0.42), *b_fs_* = −0.49, *p* < 0.001). The total effect of BA on anxiety symptom severity was significant (*b* = −0.55, 95%CI (−0.81,−0.29), *c_fs_* = −0.42, *p* < 0.001), as well as the direct effect (*b* = −0.34, 95%CI (−0.56, −0.11), *c’_fs_* = −0.26, *p* = 0.005). The indirect effect of DT on the relationship between BA and anxiety symptom severity was significant (*b* = −0.22, 95%CI (−0.39,−0.06), *ab_fs_* = −0.16), with 29.09% of the total proportion mediated.

The sixth analysis examined whether BA mediated the relationship between DT and anxiety symptom severity, per caregiver-report, while including sex as a covariate (see Figure 2). Significant associations emerged between DT and BA (*b* = 0.32, 95%CI (0.11,0.53), *a_fs_* = 0.32, *p* = 0.004) and between BA and anxiety symptom severity (*b* = −0.34, 95%CI (−0.57, −0.11), b*_fs_* = −0.26, *p* = 0.005). The total effect of DT on anxiety symptom severity was significant (*b* = −0.75, 95%CI (−0.97,−0.52), *c_fs_* = −0.57, *p* < 0.001), as well as the direct effect (*b* = −0.64, 95%CI (−0.87,−0.42), *_c𠄙fs_* = −0.49, *p* < 0.001). The indirect effect of BA on the relationship between DT and anxiety symptom severity was also significant (*b* = −0.11, 95%CI (−0.22,−0.03), *ab_fs_* = −0.08), with 14.67% of the total proportion mediated.

#### Adolescent self-report

The seventh analysis examined whether DT mediated the relationship between BA and anxiety symptom severity, per adolescent self-report, while including sex as a covariate (see Figure 2). Significant associations emerged between BA and DT (*b* = 0.64, 95%CI (0.42,0.862), *a_fs_* = 0.62, *p* < 0.001) and between DT and anxiety symptom severity (*b* = −0.64, 95%CI (−0.90,−0.38), b*_fs_* = −0.50, *p* < 0.001). The total effect of BA on anxiety symptom severity was significant (b = −0.80, 95%CI (−1.03, −0.57), *c_fs_* = −0.61, *p* <0.001), as well as the direct effect (*b* = −0.40, 95%CI (−0.66, −0.14), *c’_fs_* = −0.30, *p* = 0.003). The indirect effect of DT on the relationship between BA and anxiety symptom severity was also significant (*b* = −0.41, 95%CI (−0.62, −0.22), *ab_fs_* = −0.31), with 51.25% of the total proportion mediated.

The eighth analysis examined whether BA mediated the relationship between DT and anxiety symptom severity, per adolescent self-report, while including sex as a covariate (see Figure 2). Significant associations emerged between DT and BA (*b* = 0.64, 95%CI (0.46,0.82), *a_fs_* = 0.66, *p* < 0.001) and between BA and anxiety symptom severity (*b* = −0.40, 95%CI (−0.66,−0.14), b*_fs_* = −0.30, *p* = 0.003). The total effect of DT on anxiety symptom severity was significant (*b* = −0.89, 95%CI (−1.10,−0.68), *c_fs_* = −0.70, *p* < 0.001), as well as the direct effect (*b* = −0.64, 95%CI (−0.90,−0.38), *c’_fs_* = −0.50, *p* < 0.001). The indirect effect of BA on the relationship between DT and anxiety symptom severity was also significant (*b* = −0.25, 95%CI (−0.46,−0.06), *ab_fs_* = −0.20), with 28.09% of the total proportion mediated.

## DISCUSSION

Findings from 100 verbally fluent autistic adolescents without intellectual disability and 100 of their caregivers show that distress tolerance (DT) and behavioral activation (BA) were negatively associated with anxiety and depressive symptom severities in autistic adolescents across both raters. Stronger associations were observed between DT and anxiety across both raters, and between BA and depression. Sex differences were observed in DT and internalizing symptom severity, but not BA, such that autistic adolescents assigned female at birth exhibited lower DT and more severe internalizing symptoms than their peers assigned male at birth. Results from exploratory mediation analyses revealed important differences in raters on the roles of DT and BA in observed pathways to internalizing symptoms. Given the cross-sectional nature of the data collected and analyzed, findings should be interpreted as preliminary and with caution. Findings may be leveraged to improve our understanding of DT and BA in the pathway to internalizing symptoms in autistic adolescents and potential intervention targets. Moreover, this work furthers our understanding of DT and BA interrelationships, an emerging area that highlights the interactive nature of both concepts which contribute to internalizing symptoms (Lass et al., 2020; Lass & Winer, 2020).

From adolescent and caregiver perspectives, DT and BA were each associated with more severe anxiety and depressive symptoms, though to varying degrees; DT may be particularly important in understanding anxiety in autism, while BA may be important in understanding depression. Across both raters, larger correlation coefficients emerged between DT and anxiety symptoms than DT and depressive symptoms, while associations between BA and depressive symptoms were stronger than associations between BA and anxiety symptoms; however, it is unknown whether these correlations are statistically distinct as this was not tested. At the same time, it is important to note that DT was moderately associated with depressive symptoms across both raters and thus, DT remains an important consideration in conceptualizing depression in autism. Similarly, BA was associated with more severe anxiety symptoms, particularly among adolescent ratings. Given strong associations between DT, BA, and internalizing symptoms across raters, an exploration of DT and BA as potential mediators of these associations was conducted.

With limited research on DT in autistic adolescents, our findings of sex differences may serve as a starting point for continued research in this area and mirror some previous work on DT in non-autistic adolescents (Daughters et al., 2009, 2013), and elevated emotion dysregulation and intolerance of uncertainty in autistic females (Cai et al., 2019; Wieckowski et al., 2020). Moreover, anxiety symptoms were more severe among autistic adolescents assigned female at birth, which mirrors findings from previous groups (Corbett et al., 2022; Hiller, Young, & Weber, 2014; May, Cornish, & Rinehart, 2014; Solomon et al., 2012). Though depressive symptoms have been found to be more severe among autistic people assigned female at birth (Schwartzman et al., 2023; Trubanova et al., 2014), sex differences in depressive symptoms did not emerge in the present study and may be accounted for by a clinical sample and/or unequal sex distributions.

Mediation analyses revealed preliminary differences between raters regarding the role of BA in the DT-depression association: BA completely mediated (i.e., 70.90%) this association per adolescent self-report, but only partially mediated (27.45%) this relationship per caregiver-report. Findings should be interpreted with caution, but it is possible that BA may be *more salient* to assessment and treatment planning for depression in autism than DT; however, longitudinal studies of BA, DT, and internalizing symptoms among autistic youth are needed to truly test this initial observation. Because BA was observed to mediate the DT-depression relationship, perhaps engaging BA may be critical to ameliorating depressive symptoms (e.g., anhedonia and negative mood) in autistic adolescents. This may be further evidenced by the finding that the indirect effect of DT on depression was not significant per adolescent self-report.

In contrast, mediation models using caregiver ratings showed that BA only partially mediated (i.e., 27.45%) the DT-depression association and DT partially mediated (i.e., 20.69%) the BA-Depression association. It is possible that caregiver ratings fail to accurately capture an adolescent’s internalized experiences (e.g., distress tolerance, depressive symptoms) and/or other unexamined variables may mediate these observed relationships from caregiver perspectives. With little research on transdiagnostic mechanisms and internalizing symptoms in autistic youth, both DT and BA were tested as mediators of observed relationships to potentially inform the design of future longitudinal studies that then may be leveraged to develop more tailored interventions. Given stronger associations between BA and depressive symptoms in adolescent self-report, clinicians are encouraged to validate, and even prioritize, the preferences of autistic adolescents in facilitating family discussions of meaningful activities and designing treatment plans. Emerging research on Behavioral Activation as an intervention for autistic adults with depression is promising (Bal et al., 2023) and our findings support an extension of this work to adolescents (Menezes et al., 2024).

Mediation analyses also revealed the potential importance of both DT and BA on anxiety in autistic adolescents. Regarding the importance of DT on the BA-anxiety association, the variance in caregiver-reported DT partially explained about one third (29.09%) of the relationship, while the variance in adolescent-reported DT accounted for over half (i.e., 51.25%) of the relationship. In terms of the impact of BA on the DT-anxiety relationships, both caregiver- and adolescent-reported BA partially explained this relationship. We found a similar rater pattern such that the indirect effects of the mediator were more pronounced for adolescent-report than caregiver-report (DT: 51.25% vs. 29.09%, BA: 28.09% vs. 14.67%), which may suggest that the adolescents’ perception of these relationships differs from caregivers, and may potentially relate to differences in their perception of value-based activities. Though preliminary, the DT-anxiety results also align with prior research findings of increased intolerance of uncertainty in autistic samples with co-occurring anxiety (Conner et al., 2022; Keefer et al., 2018), as well as illustrative of the relationship of some autistic characteristics (i.e., need for sameness) and ER in anxiety symptoms (Rodgers et al., 2012). Although all anxiety models were significant, the models with DT as a mediator explained a greater proportion of the variance than those with BA as a mediator. Though preliminary, this pattern of findings may provide potential insight into how these mechanisms may relate to one another, which may guide treatment planning with evidence-based strategies for bolstering intervention outcomes for autistic children and adolescents with anxiety. Further research investigating the components of DT, including how to separately measure them and focus on them in treatment, is needed.

Based on the preliminary findings here, clinicians *targeting anxiety* in verbally fluent autistic adolescents without intellectual disability may first build DT skills (i.e., coping strategies, breathing, grounding techniques, mindfulness) in adolescents before focusing on BA-related events (i.e., exposures, social skills practice). This approach is common in several evidence-based practices (i.e., Coping Cat (McNally Keehn et al., 2013), Facing your Fears (Reaven et al., 2012)). Moreover, DT skills are core to several third-wave approaches targeting emotion dysregulation (Bemmouna et al., 2021; Conner et al., 2019; Ritschel et al., 2022), which is a key marker of anxiety in autistic individuals (Conner et al., 2020; White et al., 2014). Although DT skills are core to most evidence-based anxiety-focused interventions, this skill may be especially important in building self-efficacy for engaging in activities that relate to anxiety in autistic individuals.

### Limitations

A significant limitation of the present study is a reliance on cross-sectional data for the mediation models, which limits our ability to determine temporal precedence (i.e., we cannot determine if BA or DT causes anxiety or depression symptoms or vice versa because all data is collected at the same timepoint). Further, the cross-sectional nature of the data might compromise the ability to ascertain which variables are operationalized as predictors, mediators, and/or outcomes. A more robust investigation of these constructs using longitudinal data is an essential extension of this preliminary work. Additionally, our sample is not representative of many autistic adolescents, as it includes adolescents with average to above-average intellectual, language, and reading/writing abilities who are predominantly White and able to access psychological services. Efforts to increase the representation of all diverse autistic adolescents in our research programs, and clinics more broadly, are an ongoing area of focus. Of note, the BADS-SF and DII have not been validated in autistic adolescents. Importantly, the RCADS-47 primarily captures worry and somatic based anxiety symptoms (rather than avoidance or other behavioral manifestations) and as anxiety may present differently in autistic adolescents (Kerns et al., 2014), it will be important for future work to examine these relationships with autism-sensitive measures of anxiety (i.e., Scahill et al., 2019). Lastly, unequal distributions of autistic adolescents by sex and gender identity limit our findings.

## CONCLUSION

Our preliminary findings suggest that these transdiagnostic concepts (i.e., distress tolerance, DT; behavioral activation, BA) are important to individualizing treatment approaches, including the *timing* of certain approaches, for anxiety and/or depression in verbally fluent autistic adolescents without intellectual disability. Low DT and BA in autistic adolescents were associated with more severe internalizing symptoms per self- and caregiver-report, particularly for adolescents assigned female at birth. Preliminary results from mediation analyses may show that BA may be more salient to assessment and treatment planning for depression in autism than DT, while DT may be important for both anxiety and depression in autistic adolescents. It will be important for future work to examine these constructs and relationships longitudinally in other autistic samples, including adult samples where mental health difficulties continue to be prevalent. Moreover, it will be important to examine whether these relationships relate to outcomes in anxiety and depression and/or predictors of treatment response. Although there is growing literature on broader emotion dysregulation in autistic individuals, related concepts such as DT and BA allow for potential specificity in risk markers and intervention targets. For example, as DT captures tolerance for several aversive constructs (i.e., uncertainty, ambiguity, frustration, negative emotion, physical discomfort; Zvolensky et al., 2010), it will be important for future work to further disentangle this construct and identify altered neurobiological mechanisms of these sub constructs as they relate to co-occurring emotional and behavioral difficulties in autistic individuals. It is possible that other features common among autistic people, such as sensory differences and alexithymia, may factor into the other DT subconstructs, as insistence on sameness/intolerance of uncertainty does. Understanding the DT profile of individual autistic clients may be helpful for identifying treatment targets and the order in which a clinician approaches goals in treatment. Similarly, further investigation of BA and avoidance in autistic adolescents, specifically rewarding and meaningful activities, is an important future direction of this work. Given close associations between reward responsivity and depression, it may be critical to further investigate these constructs in this population characterized by distinct reward responsivity and high prevalence rates of depression.

## Supplementary Material

Supplemental Tables

## Figures and Tables

**TABLE 1:** Demographic information of adolescents and caregivers.

	Adolescents	Caregivers
	*M* (SD), Range	*M* (SD), Range
Age	13.70 (2.23), 10–18	45.27 (7.19), 32–78
Sex assigned at birth	61 Male 39 Female	10 Male 90 Female
Gender	61 Cisgender boy 26 Cisgender girl 6 Non-binary 5 Transgender 0 Gender diverse 2 Gender neutral 0 Prefer not to say	9 Cisgender man 80 Cisgender woman 2 Non-binary 3 Transgender 0 Gender diverse 0 Gender neutral 6 Prefer not to say
Ethnicity	10 Hispanic/Latinx 90 Not Hispanic/Latinx	6 Hispanic/Latinx 94 Not Hispanic/Latinx
Race	80 White 7 Black/African American 0 Asian 1 American Indian/Alaska Native 0 Native Hawaiian/Pacific Islander 4 Biracial 5 Multiracial 0 Other 3 Prefer not to say	85 White 6 Black/African American 0 Asian 0 American Indian/Alaska Native 0 Native Hawaiian/Pacific Islander 1 Biracial 2 Multiracial 0 Other 6 Prefer not to say
Medication status	76 Taking psychiatric medications 24 Not taking medications	
Previous counseling	78 Had previous counseling 22 Had no previous counseling	
Previous psychiatric hospitalization	30 Previous hospitalization 70 No previous hospitalization	
Aggression to self	35 Current aggression to self 65 Without current aggression	
Aggression to others	28 Current aggression to others 72 Without current aggression	
SRS-2 Total T-score	73.61 (11.68), 60–90	
Highest education		17 High school/GED 45 College degree 22 Master’s degree 6 Doctoral degree 10 Prefer not to say
Employment status		1 Student 10 Part-time employment 56 Full-time employment 22 Homemaker 11 Prefer not to say
Annual household income		16 at $25–$50,000 17 at $50–$75,000 9 at $75–100,000 13 at $100–$125,000 32 at $125,000+ 13 Prefer not to say

**TABLE 2:** Results from Spearman rho correlations of adolescent and caregiver ratings of demographic and study variables.

		1	2	3	4	5	6	7	8
1	Age								
2	DT^a^ (Adolescent)	0.04							
3	BA^b^ (Adolescent)	−0.11	0.56***						
4	Depression^c^ (Adolescent)	0.04	−0.51***	−0.65***					
5	Anxiety^c^ (Adolescent)	0.16	−0.70***	−0.63***	0.66***				
6	DT^a^ (Caregiver)	−0.14	0.27*	0.18	−0.25*	−0.24*			
7	BA^b^ (Caregiver)	0.03	0.07	0.28*	−0.33**	−0.16	0.32**		
8	Depression^c^ (Caregiver)	−0.07	−0.28*	−0.39**	0.47***	0.37**	−0.42***	−0.53***	
9	Anxiety^c^ (Caregiver)	0.07	−0.39**	−0.32**	0.34**	0.41***	−0.61***	−0.46***	0.58***

a Ratings of distress tolerance on the Distress Intolerance Index (DII).

b Ratings of behavioral activation on the Behavioral Activation for Depression Scale-Short Form (BADS-SF).

c T-scores from the Revised Children’s Anxiety and Depression Scale, Child and Parent [Caregiver] Versions.

* *p* < 0.05;

** *p* < 0.01;

*** *p* < 0.001.

## Data Availability

The data that support the findings of this study are available from the corresponding author upon reasonable request.
