# Supplementary material for: The relationship between distress tolerance and behavioral activation on anxiety and depression symptomatology in autistic youth: Leveraging self and caregiver perspectives
Source: Autism Res. Author manuscript; Available in PMC 2025 Jan 2. (PMC11695078; doi:10.1002/aur.3208)
Supplement: Supplemental Tables [file NIHMS2042263-supplement-Supplemental_Tables.docx]

**Supplemental Table 1.** Group-level differences in ratings of distress tolerance, behavioral activation, and internalizing symptom severities by autistic youth and their caregivers.

|  | **Youth Ratings**  ***N*=100** | **Caregiver Ratings**  ***N*=100** | **Test statistic** |  |
| --- | --- | --- | --- | --- |
|  | *M (SD), Range* | *M (SD), Range* | *t* | *p* |
| Distress tolerance^a^ | 28.72 (11.63), 10-50 | 29.22 (10.17), 10-50 | 0.34 | 0.74 |
| Behavioral activation^b^ | 26.56 (11.31), 6-54 | 30.65 (10.34), 10-54 | 2.76 | <.01 |
| Depressive symptoms^c^ | 63.76 (12.27), 31-80 | 68.15 (11.08), 38-80 | 3.26 | <.01 |
| Anxiety symptoms^c^ | 57.62 (14.56), 28-80 | 64.98 (13.24), 35-80 | 4.48 | <.001 |

^a^Ratings on the Distress Intolerance Index (DII); ^b^Ratings on the Behavioral Activation for Depression Scale-Short Form (BADS-SF); ^c^T-scores from the Revised Children’s Anxiety and Depression Scale, Child and Parent [Caregiver] Versions.

**Supplemental Table 2.** Results of ICC calculations using double-rating, one-way random-effects model for all variables.

| Average measures | Intraclass Correlation | 95% Confidence Interval | | F Test with True Value 0 | | | |
| --- | --- | --- | --- | --- | --- | --- | --- |
|  |  | Lower Bound | Upper Bound | Value | df1 | df2 | Sig |
| Distress tolerance^a^ | 0.444 | 0.133 | 0.644 | 1.799 | 99 | 100 | 0.005 |
| Behavioral activation^b^ | 0.367 | 0.012 | 0.595 | 1.579 | 99 | 100 | 0.022 |
| Depressive symptoms^c^ | 0.569 | 0.337 | 0.721 | 2.322 | 99 | 100 | <.001 |
| Anxiety symptoms^c^ | 0.492 | 0.218 | 0.670 | 1.969 | 99 | 100 | 0.001 |

^a^Ratings on the Distress Intolerance Index (DII); ^b^Ratings on the Behavioral Activation for Depression Scale-Short Form (BADS-SF); ^c^T-scores from the Revised Children’s Anxiety and Depression Scale, Child and Parent [Caregiver] Versions.
